# Older adults' perceptions of the risks associated with contemporary gambling environments: Implications for public health policy and practice

**DOI:** 10.3389/fsoc.2023.1061872

**Published:** 2023-03-16

**Authors:** Hannah Pitt, Simone McCarthy, Samantha L. Thomas, Melanie Randle, Sarah Marko, Sean Cowlishaw, Sylvia Kairouz, Mike Daube

**Affiliations:** ^1^Institute for Health Transformation, Faculty of Health, Deakin University, Geelong, VIC, Australia; ^2^Faculty of Business and Law, University of Wollongong, Wollongong, NSW, Australia; ^3^Department of Psychiatry, The University of Melbourne, Parkville, VIC, Australia; ^4^Department of Sociology and Anthropology, Concordia University, Montreal, QC, Canada; ^5^Faculty of Health Sciences, Curtin University, Perth, WA, Australia

**Keywords:** gambling, public health, older adults, risk environments, qualitative

## Abstract

**Introduction:**

Rapid changes in the Australian gambling environment have amplified the risks for gamblers and pose significant threats to public health. Technological advances, saturation of marketing, and the embedding of gambling in sport have all contributed to significant changes in the gambling risk environment. Older adults have witnessed the changes to the way gambling is provided and promoted in public spaces, but little is known about how these changes have shaped the way they conceptualize the risks associated with gambling.

**Method:**

Guided by critical qualitative inquiry, semi structured interviews were conducted with 40 Australian adults aged 55 years and older, who had gambled at least once in the last 12 months. Reflexive thematic analysis was used to interpret the data.

**Results:**

Participants discussed gambling environments in Australia and how they had changed through the proliferation of gambling products, environments, and opportunities; the risks posed through the embedding of gambling in community and media environments; the role of technology in gambling environments; and the role of marketing and promotions in the changing gambling environments. Participants recognized that these factors had contributed to gambling environments becoming increasingly risky over time. However, despite the perception of increased risk, many participants had engaged with new gambling technologies, products, and environments.

**Discussion:**

This research supports the adoption of public health responses that include consideration of the environmental, commercial, and political factors that may contribute to risky gambling environments.

## Introduction

Gambling is inherently about risk. As Orford (2019, p. 91) argues, the availability of self-exclusion mechanisms for gambling products and companies highlights that engaging in the consumption of these products is not an ordinary purchaser–provider activity, and there is the potential for gambling products to create addiction. Rapid technological advances, saturated and innovative marketing techniques, and the embedding of gambling within culturally valued activities such as sport have all contributed to the rapid normalization of gambling (Thomas et al., 2018). The last years of the twentieth century also saw a “*staggering liberalisation and expansion of opportunities to gamble around the world”* (Orford, 2010, p. 3). However, national gambling policies have largely failed to keep pace with the significant transformations in the gambling industry—across both land based and online products (Järvinen-Tassopoulos, 2022). Technological innovations coupled with poor regulation have made products more profitable for the gambling industry, and have significantly amplified the risks for gamblers (Deans et al., 2016), including through the exploitation of certain population subgroups (Markham and Young, 2015). These rapid changes in the gambling environment have led many researchers to argue that the contemporary gambling landscape poses significant risks to public health (van Schalkwyk et al., 2022).

While there have been significant changes in gambling environments, studies have still largely adopted a rationality approach to understanding gambling risk (Hing et al., 2019; Bagot et al., 2021). Such approaches involve a focus on the individual and the choices or decisions they make to manage predefined risks (Heidenstrøm, 2022). While these individual perspectives can provide “*important insights into the micro-level process of everyday experiences,”* they do not typically acknowledge that people's experiences and conceptualizations of risk are “*bound up within social and material structures and that individuals are positioned within a social world”* (Heidenstrøm, 2022, p. 241). Although technology has enabled the rapid transformation of gambling environments and the increased exposure of individuals to gambling products and promotions, presently, few studies have looked at these contexts through a public health or sociological lens. This includes how different population subgroups negotiate changes in the gambling environment. Gambling infrastructure has become embedded in social, symbolic, and physical environments (Thomas et al., 2023) which represents a source of risk for individuals or groups, such as the effect of increased accessibility on frequent gambling behavior (Egerer and Marionneau, 2019; Thomas et al., 2022).

Sociological and public health approaches to gambling risk environments draw attention to the complex and nuanced features of both physical and online spaces which influence the risk perceptions and behaviors of groups and individuals. Such approaches also highlight the increased potential of harm being experienced within these environments (Rhodes, 2002), and illustrate how they influence the way that risk is experienced, situated, and managed (Olofsson et al., 2019; Zinn, 2019). Such considerations also encompass how products are developed, marketed, and offered in different spaces, the discourses that frame the social and cultural acceptability of gambling, and the regulations or policies that determine and reflect the level of risk that governments consider to be acceptable for their communities (Deans et al., 2016; McCarthy et al., 2021; Marko et al., 2022). Improved understanding of the complex nature of risk associated with gambling environments and contexts, how this changes over time, and how people negotiate and conceptualize this risk in their everyday lives (or social realms) is critical to informing successful policy responses to regulating gambling risk environments (Zinn, 2019).

Understanding the socio-cultural dimensions of risk environments is also important for shifting dominant discourses and policy approaches that have stubbornly focused on individualized framings of responsibility for gambling risk and harm (Marko et al., 2020), and marginalize the risks attributable to gambling products and environments (van Schalkwyk et al., 2021a). Public health approaches are important because they move beyond a view of harm that is entirely the responsibility of the individual and focus also on situations and environments in which risk and harm is constructed (Marko et al., 2022; Nyemcsok et al., 2022). This invites consideration of the environmental and commercial factors that influence pathways to gambling (Bestman et al., 2020), including risky behaviors that occur within these contexts. Such studies are common in other areas of public health—for example in the field of alcohol where there is recognition that the environments in which drinking occurs can have a greater influence on behavior than individual characteristics (Kairouz and Adlaf, 2003; Shortt et al., 2018). However, in relation to gambling, relevant research has focused mainly on understanding individualized gambling behaviors, which has led to recommendations for individually-focused harm minimization strategies (Gibbs Van Brunschot, 2009; Fontaine et al., 2021). These types of responses may be convenient for policymakers because they provide a set of discrete responsibility behaviors and are able to be easily translated into a set of policy responses that are likely to be favored by governments and the gambling industry. However, such approaches fail to address the broader structural, environmental, and political factors that contribute to gambling risk and harm (McCarthy et al., 2021; van Schalkwyk et al., 2021b).

Older adults have been identified as one such group that may be particularly vulnerable to the changing gambling environment (Guillou Landreat et al., 2019; Fontaine et al., 2021) and have witnessed significant changes in the gambling environment over time. Researchers have explored how socio-cultural and commercial factors may interact to make community gambling venues and casinos popular and potentially risky spaces for older adults (van der Maas et al., 2017; McCarthy et al., 2021; Pitt et al., 2021). Relevant factors that may have unique impacts on older adults include the high accessibility of gambling products and venues in local communities and perceptions of venues as safe and accepting social spaces; non-gambling inducements that may encourage older adults to attend venues, including free transport, discounts on meals; and loyalty schemes (McKay, 2005; Dyall et al., 2009; Thomas et al., 2020; McCarthy et al., 2022). However, there has been little research that has qualitatively explored older adults' attitudes and reflections of gambling environments and how these environments may contribute to their perceptions of the risks relating to gambling.

The following study aimed to explore how older adults conceptualized the changing gambling environment in Australia, and the impact that changes had made on their perceived risks associated with gambling. The study was guided by three research questions:

1. Have older adults observed any changes within the Australian gambling environment?
2. How do older adults conceptualize any changes they have observed in the Australian gambling environment?
3. Do older adults perceive that any changes in the gambling environment are associated with any subsequent alterations in the risk of gambling harm?

Understanding how individuals conceptualize and interact with gambling environments is important in developing public health strategies that move away from a predominant focus on individual actions and responsibility, and toward the social situations, structures, and environments that may influence risk behaviors in different population subgroups (Rhodes, 2009). We subsequently consider the implications of the study findings for public health policies that aim to reduce the risk of gambling harm for older adults.

## Methods

### Approach

This research was part of a larger study that explored the normalization of gambling for older adults in Australia. One other paper has been published from this study exploring the gambling practices of older adults (Johnson et al., 2022). The current study took a critical qualitative approach to inquiry (Denzin, 2017), which thus focused on the role of power, privilege, and inequality in health and social issues (Charmaz, 2017; Jacobson and Mustafa, 2019), challenging assumptions, and identifying points of intervention for social and policy change (Cannella et al., 2016). As Fielding (2020, p. 143) states, qualitative research is impactful for considerations of policy reform, as it can “*reveal unknown or unexpected things about the social world*.” Ethical approval was obtained from the Deakin University Human Research Ethics Committee (2019-354).

### Sampling and recruitment

Participants were required to be aged 55 years or over, a resident in Australia, able to speak English at a level that would allow them to comfortably participate in the interview and have gambled on at least one gambling product in the last 12 months. The age range of 55 years and above was chosen because it is comparable to other gambling studies with older adults (van der Maas et al., 2017; Granero et al., 2020; Rockloff et al., 2020), and ensured a range of gambling experiences associated with working, retirement, and aging were captured. While most studies of gambling harm focus on individuals who already engage in gambling at problematic levels, the study aimed to sample participants experiencing no, low, or moderate levels of risk associated with gambling. Researchers have outlined that while many older adults report that they engage in gambling for leisure and entertainment, harms still occur across the risk spectrum, with very little known about this group as compared to people with significant gambling problems (Hilbrecht et al., 2020).

Convenience sampling techniques were used to recruit participants through various networks, including via local councils, through community organizations, on social media sites, and through a database maintained by the research team that included individuals who had consented to be contacted for future research studies. Snowball sampling methods were also used whereby participants were asked to share the study information with their networks or partners. Individuals were sent a Plain Language Statement with more information about the study and gave written or verbal consent before participating in the interview. Participants received a $50 grocery voucher as a token of appreciation for their time.

### Data collection

Semi-structured interviews lasting approximately 1 hour were conducted via telephone over a 6-month period between July 2020 and January 2021. Interviews were audio-recorded and professionally transcribed. Most participants completed the interview on their own, while four participants (two couples) chose to participate together. Participants were asked demographic questions about their age, state of residence, occupation, and education, as well as questions relating to gambling risk from the nine-item Problem Gambling Severity Index (PGSI) (Ferris and Wynne, 2001). Open-ended questions then prompted discussions about perceptions of gambling environments, the risks posed by these, and how participants conceptualized this risk. Interviews continued until there was significant breadth and depth in the responses to construct an analytical narrative with enough information power to answer the research questions (Malterud et al., 2016; Braun and Clarke, 2021b).

### Data analysis

Data were interpreted using Braun and Clarke's (2006, 2021a) six steps of reflexive thematic analysis. The first step involved familiarization with the data, including reading and re-reading the transcripts and listening to the audio-recordings. The coding process then involved open coding techniques, with data coded across transcripts in relation to the study research questions. The coding first looked at the semantic or surface meanings in the participant responses, and then explored the latent or implicit meanings in relation to experiences and perceptions of gambling risk environments. Codes with similar meanings were grouped to construct initial themes about such environments, which were then refined with the broader group of authors to ensure they reflected the study aims and research questions. The researchers continuously reflected on the codes and themes in relation to the whole data set, which ensured the themes were true to the data and demonstrated a pattern of shared meaning. Each theme was constructed and defined, with the analytic narrative for this paper focusing on older adults' perceptions of the modern gambling environment, placing emphasis on participant perspectives about how changes in the environment may have influenced their own gambling risk perceptions and behaviors. In accordance with reflexive thematic analysis, the research team met regularly to discuss the developing themes and reflect on any assumptions that might have shaped the theme development and the interpretation of the data.

## Results

### Participant characteristics

A total of *n* = 40 older adults participated in this study, with the sample characteristics presented in Table 1. Participants were aged between 55 and 78 years (*M* = *65.7*, SD: 6.7), with most residing in the state of Victoria (*n* = 21, 52.5%). Slightly more females (*n* = 23, 57.5%) participated in the study than males, and two-thirds of all participants were retired (*n* = 26, 65.0%). The sample did not include any problem gamblers, although most participants were at risk of gambling harm, with 19 older adults (47.5%) classified as low-risk gamblers and six (15.0%) classified as moderate-risk gamblers.

**Table 1:** Sample characteristics.

**Characteristic**	***n***	**%**
**Gender**		
Female	23	57.5
Male	17	42.5
**Age**		
55–59	10	25.0
60–64	5	12.5
65–69	12	30.0
70+	13	32.5
**Occupation**		
Retired	26	65.0
Working	14	35.0
**Education**		
University	19	47.5
Did not complete high school	10	25.0
TAFE^*^	7	17.5
Completed high school	4	10.0
**Geographical areas**		
Victoria	21	52.5
New South Wales	15	37.5
Queensland	3	7.5
South Australia	1	2.5
**Gambling status (PGSI score)**		
Non-problem gambler (0)	15	37.5
Low risk (1–2)	19	47.5
Moderate risk (3–4)	6	15.0

* TAFE: Technical and Further Education.

Four themes were constructed from the data.

### A proliferation of gambling products, environments, and opportunities

Participants discussed how gambling environments in Australia had significantly changed over the years. The most common observation was in relation to the accessibility and availability of gambling. For example, some recalled a time when gambling was restricted in community settings. This included references to the types of gambling products that were available, the environments in which they were available, and the time of day when they were available. Some participants specifically commented on the changes in the provision of electronic gambling machines (EGMs or poker machines—“pokies”) in community settings. They reflected that EGMs were once difficult to access, because there were government regulations that prevented them from being made available in certain Australian states. For example, some participants noted that EGMs were not previously available in pubs, and there were no local casinos, meaning that people could not easily engage in gambling on the products. For example:

When I was young there was only one casino in all of Australia, so you couldn't just pop down to the local casino.–56-year-old female, non-problem gambler.

Geographical restrictions and less sophisticated technological features (relative to modern gambling environments and products) meant that gambling was a less convenient and regular activity that individuals could engage in. Participants reflected that they had to travel long distances to engage in gambling, with some Victorian participants explaining that they had traveled interstate to New South Wales to gamble on EGMs, or to visit a casino. Participants also reflected that historically, gambling was rarely visible within their local communities, stating that the opening up of different geographical locations for gambling may have originally made economic sense to governments, but now has created new risks and harms for the community:

Originally when we were younger, we had to go to New South Wales to play. You'd go and play a poker machine, you'd never see them here. But then we thought well, if Victoria licenses them and makes them, it will keep the money back here in Victoria and the government will have more money to spend on whatever they spend it on and it will save it going out of the state. But I can see now that it causes more problems than any good it does.–70-year-old male, non-problem gambler.

Participants commented on how specific changes in the regulation of gambling contributed to round-the-clock and seven day a week continuous availability of gambling products. Some commented that regulatory changes also meant that individuals now rarely had to travel far to gamble and could gamble on a range of products in their own homes. The removal of the geographical barriers to gambling was described as moving gambling away from being “*a novelty thing”* to one that was “*readily available.”* Some participants reflected that this also meant that previous boundaries around when and how much individuals could gamble had largely disappeared. There were reflections on how this had amplified the risks of gambling. Comments such as “*it's probably over the top,”* were used to describe the excessive proliferation of gambling opportunities and the risks that these created:

If it's easy to do and you've really got permanent access it makes it tempting. More temptation.–60-year-old male, low risk gambler.

Some participants also commented that increased accessibility and availability had contributed to the shift from gambling being a social activity to one that was increasingly related to individuals feeling like they were being encouraged to gamble. Some specifically commented that while increased accessibility made gambling, and specifically betting, more engaging, it also made gambling a riskier activity. Several men in particular spoke about previously being able to wager on sports and horses through land-based venues only, such as through bookmakers at racetracks or at the local TAB betting shop. One man discussed that having to attend gambling venues was a barrier to instantaneous gambling and suggested that this did not exist today. Others also highlighted the ability to gamble anywhere, at any time, on events around the globe:

Oh, yeah. Like I said to you before, you bet online, bet through your app. More race days or seven days a week. It is basically 24 h a day racing and I think as soon as local racing finishes late at night at the greyhounds, then you've got all the overseas racing out of Europe. And then in the morning, out of America.–60-year-old male, low risk gambler.

### The risks posed through the embedding of gambling in community and media environments

When discussing the changing gambling landscape over time, some participants particularly referred to the way in which gambling has become embedded in everyday community settings. Some noted that they now regularly walked past gambling products and venues in their local communities, including lottery outlets in shopping centers and EGMs in venues that they went to for entertainment or meals. Many of these participants commented that the presence of gambling in everyday settings and the alignment with non-gambling activities led to increased engagement in gambling. For example, one participant felt that having gambling products embedded in environments where there was alcohol created a “*trap”* for risky gambling, and contributed to gambling being perceived as socially accepted:

I think we've normalized it to the point where if you go shopping you can buy a scratchy or if you—you know like it's okay to go out for a meal and go and gamble. It's part of that normal social experience that we actually have.–62-year-old male, low risk gambler.

A few participants commented that if gambling products were not as available in local communities they would be used less frequently—“*you wouldn't go out of your way looking for it.”* Some stated that because gambling was “*easy”* to access it was a more “*tempting”* activity, while the availability of gambling, combined with commercial motives of the gambling industry and provision of multiple opportunities to gamble, made gambling riskier:

I mean, the thing with gambling these days is it's just pervasive. It's everywhere. When I was younger, you used to bet on the horses or the dogs or something like that. But now, you've got football, politics, you can bet on who's going to win *The Voice*. All these companies making all these free offers and stuff like that. I think it's a real trap, really. And you can go anywhere now. In the old days, you'd to have to go to the TAB. But now, you just go to your local pub or you do it on your phone. It's a real trap.–66-year-old male, moderate risk gambler.

There was additional concern that the embedding of gambling venues within local communities had exacerbated the vulnerabilities in communities that were already experiencing disadvantage. For one participant, this created a sense of moral discomfort in relation to the location of EGM venues in lower socio-economic communities:

To see clubs in less privileged areas of Sydney out in the west very heavy with immigrants, to see these... I mean, the really well fitted out clubs in these low-income areas and you just go, “Yeah, I know where that money came from,” and that doesn't really feel good.–57-year-old female, low risk gambler.

Gambling was also embedded in everyday lives through the media. For example, a few participants commented that televised lottery draws had initially brought gambling inside the home, while one woman suggested that lotteries “*became this big event”* which led people to believe that they could win amounts of money that would change their life. The following participant attributed televised lottery draws to significant shifts in how people viewed the benefits of gambling:

I think that that changed something in the psyche of people. They felt that they could really, with one ticket, win a really big amount of money and change their life.–73-year-old female, low risk gambler.

Another considered that watching the lottery broadcast was an event for the whole family. People wanted to join the “*party like atmosphere”* that had been created around the lottery draw:

Once upon a time, I don't know if they still do it, but on Saturday night they would have the big thing where all the balls dropped. You would see all the cultured balls dropping and people would sit there waiting for their numbers to fall. And there'd be balloons and it was a very party-like atmosphere. And I think most people are the same. You see a party you'd like to join in. And then they made a big deal about, you see the man going to deliver them their cheque. It was all very lovely.–72-year-old female, non-problem gambler.

For many participants, the closure of gambling venues during the COVID-19 pandemic restrictions enabled them to reflect on how much the embedding of gambling in their everyday lives had contributed to gambling risks. However, despite social distancing restrictions, which had led to the closure of many land-based gambling venues, and the unwillingness of participants to visit venues due to the perceived health risks, there were still opportunities to gamble:

**Participant:** Well, I don't have to go and gamble and you could always put some money in the TAB account online if you wanted to have a gamble on the horses. So you can do all that without having to go out into the community.

**Interviewer:** You weren't too bothered by the venues being closed?

**Participant:** No, not really. Only that I had to learn how to transfer the money into that account. But I've got it down pat now.–63-year-old female, non-problem gambler.

### The role of technology in risky gambling environments

Technology was perceived as playing a powerful role in the creation of risky gambling environments and was one of the biggest changes participants had observed over time. It made gambling instantaneously accessible and as easy as “*clicking a button on their app*.” Many participants observed how these changes in technology had also altered their own engagement with gambling. Several participants indicated that changes in the design of some gambling products had increased the risks associated with gambling, which had led to a reduction in their own gambling behaviors. These participants said that they now rarely engaged with certain products because they did not understand how they worked, and were concerned about losing a lot of money:

**Participant:** I don't really understand the electronic poker machines. Back in the days when they used to be called the one arm bandits, and you used to have to jiggle their arm and see if you had some influence over the machine.

**Interviewer:** So how do you feel about them now?

**Participant:** I probably rarely go near them. You just press a button. Like oh, yeah, great. I guess it's quite easy to lose a lot of money very quickly now. Because how often does a spin occur and how often can you press that button?–59-year-old male, non-problem gambler.

However, there were other participants who described embracing these new technologies. They explained that they felt very “*tech savvy*,” and had personally taken up new technologies, including downloading gambling apps on their phones. While there was limited choice in the past, some participants perceived that developments in technology meant people could now gamble on a broader range of products and events. This had evolved from betting on outcomes of sporting matches to being able to bet on “*who scores the first goal or when did the first wicket fall.”*

Some participants described how new technologies had also moved gambling to a more individualized activity. They commented that gambling had been largely transformed from something that was socially done with friends to something that encouraged individuals to gamble alone when they otherwise would not have done. Participants perceived that these new technologies had contributed to individuals gambling more money, and more frequently than they normally would. This was not only in relation to wagering, but also to other products such as lotteries. Women perceived that these new technologies created more risks for young rather than older generations:

You can even print off the TattsLotto tickets, like five TattsLotto tickets online. You don't even have to print it off, but yeah, so I think definitely technology's a great influence on, I think the amount and frequency that a certain demographic, again, we're probably talking about a younger demographic that would've been increased. When I was in that demographic, that's the probably 20s, people, they might've placed a bet occasionally on the weekend, on a race or specific races. But now that demographic bets on a whole lot more.–66-year-old female, non-problem gambler.

Some participants noted specifically that it was rare to see young people in traditional land-based betting venues, suggesting that other environments now appealed to these subgroups:

You rarely see anyone under 35 come in (to the TAB). And if they do, they'll stay 2 or 3 min. But when you talk to some of them including some of my nephews and that, the vast majority have got phone apps and they've bet on sport, including some of the American sports. Gridiron and basketball and stuff.–60-year-old male, low risk gambler.

Participants also commented on how technology had linked betting with banking, again transforming gambling environments. The link between using apps to gamble and online banking meant that money could be moved into the app easily, which was perceived to create additional risks as individuals could spend more money than intended. This contrasted with previous experiences of only having access to the money brought to the gambling venue:

In the old days, you'd have to go to the races or go to an actual TAB or get on the phone and talk to an operator and place your bet. Now, you can pick up your mobile, you've got the app on your phone or if you haven't, you download it. You do it and even if you run out of money you can transfer money from your bank account over to your betting account to keep betting if you want to or use your credit card where you're paying cash advance fees on drawing the money and interest from day one, which I've seen people do before. It's a lot more accessible, a hell of a lot more.–62-year-old male, non-problem gambler.

### The role of marketing and promotions in the changing gambling environments

Finally, gambling-related marketing and promotions were commonly referenced by participants and contributed to a perception that gambling was “*in your face a lot more”* than in previous years. Participants described the “*proliferation”* of gambling advertising in popular and high traffic areas, as well as an influx of promotions on television. Older adults described gambling advertising as being “*all over the TV all the time.”* Some were concerned that the volume of advertisements reflected attempts to “*suck”* people in and created a riskier environment in which advertising influenced more people to engage in gambling than in previous years. Others commented that people were more socially tolerant of gambling advertising, with one man stating, “*if you tried to have advertising like they do now 30 years ago there'd probably be hysteria*.” Participants reflected on their own childhoods, commenting that changing media environments contributed to the intensity of gambling marketing:

It's more intense, I think. You know, when I was a child, I never ever knew that people did this sort of thing. Not that I read many newspapers as a child but, you know, there was no television either, so…But you know, even watching sport on TV, they'll sort of advocate that you can gamble on it. And I think that it has probably increased, with people thinking it's normal to just do that.–78-year-old female, non-problem gambler.

Some participants spoke about the content of gambling advertising shaping positive attitudes and referenced the glamor associated with horse racing events and the link with sports and athletes as creating a perception that engaging in gambling was a normal activity. Others commented that the promotion of large lottery wins, and jackpots also influenced individuals to “*have a go and try to win some of that.”* Some participants were critical of how gambling was represented in advertising, stating that the reality of gambling outcomes did not match what was represented in relevant marketing. For example, one man described how the marketing of horse racing made it look like a “*beautiful place”* compared to the reality of “*drunken people in bins spewing their guts and laying all over the place*.” Others were critical of the way that advertising created the perception that winning was easy and common, and did not provide an accurate representation of the negative outcomes or risks associated with gambling:

They try to make it seem exciting, fun. Like it's usually they show a group of blokes in a pub, and how they win, and they high five, and everything, and they seem to be having huge amounts of fun and always winning. You never see it where they show, “Oh, we lost. Oh, can't afford to have a drink.”–73-year-old male, non-problem gambler.

## Discussion

This study aimed to improve understanding of the changing gambling risk environments from the perspectives of older adults. While there has been significant and legitimate concern about young people and gambling, this should not preclude recognition of the impacts on other age groups who may be vulnerable to gambling harm for different reasons. We sought to understand how older adults observed changes in gambling environments and associated risks, including how changes in these environments had impacted on their own attitudes and behaviors. The findings raise a number of points for discussion in relation to gambling risk environments, and the public health strategies that could be used to respond.

The participants in this study described profound changes in the Australian gambling environment over time, including trends for gambling products, spaces, and promotions becoming more convenient due to rapidly changing technology, and the embedding of gambling into everyday spaces. These narratives highlight a recent time when gambling products and promotions were not considered normal, everyday occurrences within Australian communities. There was clear acknowledgment from older adults that modern gambling environments (including technologies and commercial marketing) create new risks for many population subgroups, mostly through the increased accessibility, availability, and social acceptance of gambling. However, despite an acknowledgment of these new and emerging risks, many participants described having adapted to these gambling environments by adopting new forms of technology that supported their own participation in gambling. This provides support for Egerer and Marionneau's (2019) argument that new gambling cultures and spaces of convenience, along with liberalized regulation of gambling, may have a significant impacts on risk, and reinforce gambling as a socially acceptable activity. It also challenges assumptions that only young people or men are vulnerable to the rapid changes in gambling technology, highlighting the important role of critical qualitative research in challenging assumptions about gambling behaviors, and providing nuanced information to guide public health policies and practice interventions (Fielding, 2020).

While the accessibility and availability of EGM venues has been shown to be a key motivator for older adults' EGM gambling (Pitt et al., 2021), and may reduce the perceived risks associated with these products (McCarthy et al., 2021), this study demonstrates that older adults engage with many different gambling products across contexts. The findings demonstrate the importance of research that considers how individuals interact with gambling structures, cultures, and environments (Heiskanen and Matilainen, 2020), and how these interactions evolve over time. Among other things, this highlights that risk is not static, and there is a need to monitor how older adults engage with different gambling environments, products, and promotions as industry strategies evolve over time. These types of studies will also help to debunk stereotypes about gambling behaviors—for example that the only environments that may pose risks to older adults are EGM venues and casinos.

Older adults' perceptions of the changing gambling environment and their concerns about associated risks, raise important areas for consideration from a public health perspective. Findings relating to increased accessibility, diversification, and promotion of gambling products align with broader discussions about how best to address the harms associated with gambling at a population level (Goyder et al., 2020; Johnstone and Regan, 2020). The evolution of commercial marketing practices is not unique to gambling; researchers have identified similar ways in which many harmful industries, such as the tobacco industry, have changed in response to attempted regulatory reforms, and provide cautionary advice that public policy must anticipate these changes and be sufficiently broad to cover the evolving market (Ling et al., 2022). In the context of liberalized gambling environments, there is a critical role for governments in designing and implementing regulations that can proactively keep pace with changes in gambling environments, products, and promotions. While risky gambling environments remain poorly regulated in many jurisdictions, including Australia, we would argue from a public health perspective that robust regulation of the contexts in which gambling occurs would provide one of the most effective measures for reducing the risks of gambling.

Perspectives on the proliferation of gambling products, opportunities, and promotions in everyday environments also calls into question the appropriate primary focus of harm minimization efforts. These have traditionally been based on rationality models of risk that focus on individual behaviors (Young et al., 2021; Dowling et al., 2022), and it seems implausible that they can adequately address the range of gambling industry strategies, structures, and environments that participants in this study recognized as contributing to risk environments. The findings from this study provide further evidence for a comprehensive public health approach to harm prevention, which can move beyond “responsible” or “safer” gambling strategies, and toward addressing the broader environmental, commercial, and political factors that may influence risky gambling behaviors.

While the behaviors of participants in this study had not yet progressed to problematic or pathological levels of gambling, we would argue that many could be susceptible to progressing down this path if they are further exposed to risky gambling environments and products. Participants in this study had not only noticed environmental and structural changes that they considered could increase the risk of gambling harm, but also described ways in which their own gambling behaviors had diversified and increased in response to these changes. This was seen, for example, among those who reported having expanded their gambling activities to include new online betting opportunities which were linked directly with bank accounts.

## Limitations

This study was limited as it recruited most participants from the states of Victoria and New South Wales in Australia. These states have distinct gambling contexts and environments compared to other Australian states, such as Western Australia, which has regulations that prevent some gambling products being accessible in community settings. Future research should explore the differences between the gambling environments across a range of jurisdictions and the perceptions of gambling risk in different contexts. Other characteristics such as gender, socio economic status, or gambling product preference may also provide valuable insights into how older adults construct attitudes about gambling risk and gambling environments. The sample did not include any individuals who were classified as having problem or pathological levels of gambling. Problem and pathological gamblers, who are at most immediate risk of gambling harm, may have varying perceptions of gambling environments, which is an important consideration for future research and public health action.

## Conclusion

This study provides new information about gambling risk environments, and how changes in these can impact on different population subgroups, including older adults. It shows that older adults' participation in gambling continues to evolve and should be subject to ongoing monitoring. The challenge for public health researchers and advocates is to convince governments to move beyond simplistic individualistic risk responses, and to focus on the range of structural factors that may contribute to gambling harm. The findings support calls for stronger regulation of risky gambling environments in order to protect vulnerable populations. Governments should implement regulation that keeps up with the changing and expanding gambling environment, and the risks that this environment poses to different population subgroups.

## Data availability statement

The dataset analyzed in the current study is not publicly available, or available on reasonable request because participants explicitly consented to only have their data shared with the immediate research team. Requests to access the datasets should be directed to hannah.pitt@deakin.edu.au.

## Ethics statement

The studies involving human participants were reviewed and approved by Deakin University Human Research Ethics Committee (2019-354). The patients/participants provided their written and verbal consent to participate in this study.

## Author contributions

HP and ST: conceptualization of the study, data analysis, drafting, and critical revisions of the paper. SMC and SM: data analysis, drafting, and critical revisions of the paper. MR, SC, SK, and MD: conceptualization of the study and critical revisions of the paper. ST, MR, SC, SK, and MD are the study chief investigators. All authors contributed to the article and approved the submitted version.
